# Conversion of Failed Hip Hemiarthroplasty to Low Friction Arthroplasty (LFA)

**DOI:** 10.3390/jcm8040503

**Published:** 2019-04-12

**Authors:** Levent Bayam, Efstathios Drampalos, Hajime Nagai, Peter Kay

**Affiliations:** 1 Orthopaedics, Sakarya University, Sakarya 54100, Turkey; 2 Orthopaedics, Manchester University Hospitals, Manchester M23 9LT, UK; edrampalos@yahoo.gr; 3 Orthopaedics, Wrightington Hospital, Wigan WN6 9EP, UK; hajime.Nagai@wwl.nhs.uk (H.N.); peter.Kay@wwl.nhs.uk (P.K.)

**Keywords:** low friction arthroplasty, conversion of hemiarthroplasty to THA (total hip arthroplasty), dislocation, failed hip hemiarthroplasty

## Abstract

**Purpose:** We aimed to study clinical and radiological outcomes of conversion from hemiarthroplasty to Charnley hip replacement (CHR) with a particular concern over reported increased dislocation rate and literature review. Conversion of hip hemiarthroplasty to total hip replacement (THR) is a procedure reported to have high rates of complications. In the literature, there is no specific study on small head conversion. The purpose of this study was to evaluate the conversion of failed hip hemiarthroplasty to CHR with the use of modern implants. **Methods:** The study included 42 patients, who underwent the above procedure. The operations were carried out using a modern Charnley-type THR with a 22-mm diameter of femoral head and a trans-trochanteric approach. The mean follow-up was 75.7 months (range 25–171). Radiographs from the last follow up were evaluated for loosening and other reasons of failure. Clinical outcome was assessed using postoperative pain, function scores, complications and implant survivorship as well as radiological evaluation. Charnley’s modified pain and mobility scoring system were used for clinical and Hodgkinson and Harris’ criteria were used for radiological assessment. **Results:** Functionally, all of the patients showed improvement. Mean improvement in the pain level was by average of 2.4. On mobility assessment, 38 patients (90.4%) improved. Three patients (7.1%) had recurrent infections and three (4.8%) cases were treated with revision surgery and pseudarthrosis. Further complications occurred in 19.1%, not requiring operative treatment. On radiological evaluation, one (2.4%) case showed cup demarcation without bone loss, two (4.8%) cup migration, and one (2.4%) stem demarcation. Kaplan Meier survival analysis showed a survival of 90% at 96 months of follow up (95% CI (confidence interval), 60–90). **Conclusion:** Larger head might not be the answer to decrease the dislocation rate. Complication rates during revision of hip hemiarthroplasty to modern CHR with 22.225-mm head diameter were comparable to first-time THR revision despite having a smaller head.

## 1. Introduction

Most cases of displaced intracapsular femoral neck fractures are seen in elderly patients and the choice of treatment is hip hemiarthroplasty [1]. Hemiarthroplasty has shown good short-term results in this group of patients (elderly patients), particularly with regards to pain relief, return to activity, morbidity and mortality even if total hip replacement (THR) may give better function in the healthiest and fittest of the elderly patients [2,3]. Another option is bipolar arthroplasty which has another interface inside the bipolar head and was considered to reduce wear, thereby, improving the long term outcome [4]. However, the studies comparing bipolar to monopolar hemiarthroplasty recently showed hardly any difference between them regarding morbidity, mortality and functional outcome [2].

Charnley’s studies led to the development of Low Friction Arthroplasty (LFA) concept and long-term follow up showed a success story with a 78% femoral stem implant survival in 35-year follow-up [5]. In the recent years, the design of total hip replacement has improved in terms of bearing surfaces and use of larger femoral heads as well as improvement in surgical techniques, and these were considered to be safer with less mechanical complications and dislocations [6].

Meanwhile, the most common reason for revision of a failed hemiarthroplasty to a THR is pain [7]. There may be different reasons of failure but most commonly it is acetabular erosion, which is as high as 66%, and femoral loosening [1]. Other reasons of failure are recurrent dislocations, protrusio acetabuli, periprosthetic fractures, and infections [8]. Conversion of a failed hemiarthroplasty into a THR is not an easy procedure. Previous studies observed a notably higher failure rate for conversion of Hemi to THA (CTHA) than for primary total hip arthroplasty (THA) revision [1,9,10,11]. The number of publications in the literature on this subject, however, is limited and there is no specific study on small head conversion.

We aimed to study clinical and radiological outcomes of a challenging group of elderly patients with conversion of hemiarthroplasty to CHR using the modern Charnley-type implants and 22.225-mm head diameter, also to find out if there is any unique problem different from other revision groups with a particular concern over increased dislocation rate and to review literature.

## 2. Methods

All the patients included to the current study underwent conversion of a failed hemiarthroplasty to CHR and mean follow up was over 6 years. We considered all hemiarthroplasties regardless of the indication for the primary operation (hip fracture, osteonecrosis of the femoral head, pathological fracture) and with a minimum of a 2-year follow-up. All operations were carried out in a single unit with a modern Charnley-type cemented THR primarily using 22.225-mm diameter femoral heads through a trans-trochanteric approach. Whenever there was acetabular erosion and cavitary defects, the impaction bone grafting technique was used.

Forty-two patients (37 females and 5 males) who underwent conversion from failed hemiarthroplasty to CHR were identified. The mean follow-up was 75.7 months (range 25 to 171). The outcome was based on function, postoperative complications, implant survivorship and radiological evaluation. Charnley’s modified pain and mobility scoring system was used to assess function. This scoring system was initially described by Merled’Aubigne and Postel in 1954 and later modified by Charnley in 1972 [12] (Table 1).

Hodgkinson and Harris’ system was used for radiological assessment [13]. With this system, a cup is considered to be loose when there is more than 1-mm of radiolucency at the cement bone interface in all three zones (Table 2).

Our unit has a high volume of hip operations and according to UK National registry, the unit performed over 1300 primary and 300 revision hip replacements yearly [14]. In the unit, the scoring systems were collected pre-operatively and postoperatively in each follow-up clinic. The clearly documented medical records were analyzed thoroughly together with well-preserved hip anteroposterior (AP) and lateral (LAT) x-ray views. Follow-up radiographs were also assessed for stem subsidence, appearance of radiolucent lines, osteolysis, stress-shielding of proximal femur, loosening, and bony ingrowth. The patients were further assessed with full blood count (FBC), erythrocyte sedimentation rate (ESR), C-reative protein (CRP) and bone scan in case of suspicion for infection.

Statistical analysis was performed using Student’s t test and Chi-squared test (SPSS version 21 SPSS, Chicago, IL, USA). Difference was considered significant at *p* value < 0.05. Kaplan-Meier estimates were calculated to describe CHR survivorship (cumulative probability of revision or conversion to pseudarthrosis) with point wise 95% confidence intervals (CIs).

The study was a retrospective study and conducted according to The Declaration of Helsinki, and it was approved by the local audit department of the Trust. The data used to support the findings of this study are available from the corresponding author upon request.

## 3. Results

Forty-two patients (37 females and 5 males) were included. The mean age at the time of the hemiarthroplasty was 65 (range 34 to 77) and at the time of CHR was 70 (range 51 to 80). The most common type of hemiarthroplasty was the cemented Thompson and followed by the Austine-Moore (Table 3). The causes for the hip fracture were mainly trauma, osteoporotic fractures and there was one case of osteonecrosis. 

Table 4 shows whole complications following hip hemi-arthroplasty before conversion to LFA. Among those, the most common indication for revision was acetabular erosion (62%) and this was followed by stem loosening (23.8%), infection (11.9%) and periprosthetic fracture (2.4%) (Table 4). Wound infections, deep vein thrombosis (DVT) and some of the deep infections were not indications for conversion. 

With regards to the acetabular erosion (26 patients), there was a tendency to erode medially when there was an infection but there was purely superior wall erosion when there was no infection. We distinguished acetabular erosion to different groups (Group A: superior wall erosion, Group B: medial wall/superior medial wall erosion) and two causes (infection or no infection) as can be seen on Table 5. Statistical analysis showed that infection was the cause of superior acetabular wall erosion in 14.2% compared to the 66% on the group of medial wall/superior medial wall erosion (*p* = 0.0216). 

Post revision, all the patients showed improvement in their pain and walking scores. Pain improved from a mean of 3.02 preop (SD = 0.72) to a mean of 5.39 (SD = 0.89) post CHR (*p* < 0.0001). Walk improved from a mean of 2.69 (SD = 0.75) pre THR to a mean of 4.64 (SD = 1) post CHR (*p* < 0.0001).

**Figure 1:**
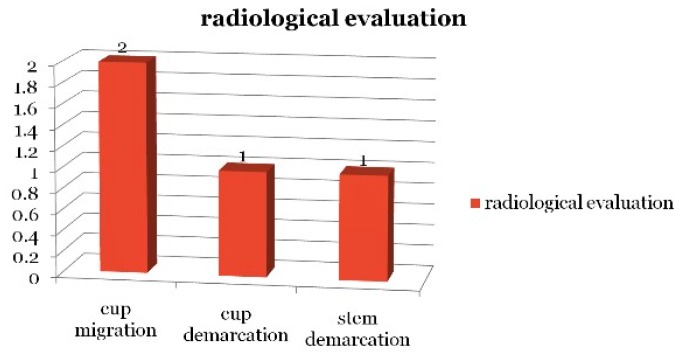
Radiological evaluation of Total Hip Replacements.

On radiological evaluation, one case showed cup demarcation without bone loss (types 1,2,3 acetabulum), two cup migrations (type 4 acetabulum), and one stem demarcation (Figure 1). 

Table 6 shows the complications following revision or conversion surgeries. Several complications occurred in these (42) patients as below and only two patients had repeat revision. Four cases sustained a postoperative dislocation (9.5%), none of which required revision. During the follow-up period, out of four dislocations, there was no recurrent dislocation and one patient needed a brace. Three cases (7.1%) showed clinical signs of resistant or recurrent infection, two wound dehiscence (4.8%), one symptomatic trochanteric non-union, and one trochanteric bursitis (Table 6). Two patients required re-revision for their hip replacements. Whilst one patient with infection had a revision to convert THA to pseudoarthrosis, the other patient had an operation following long-term antibiotics; the operation included removal of the implant, sinus excision and conversion of pseudo to LFA. There was one patient with wound infection had an operation for herniation due to fascia defect. One patient with trochanteric bursitis had removal of wires.

**Figure 2:**
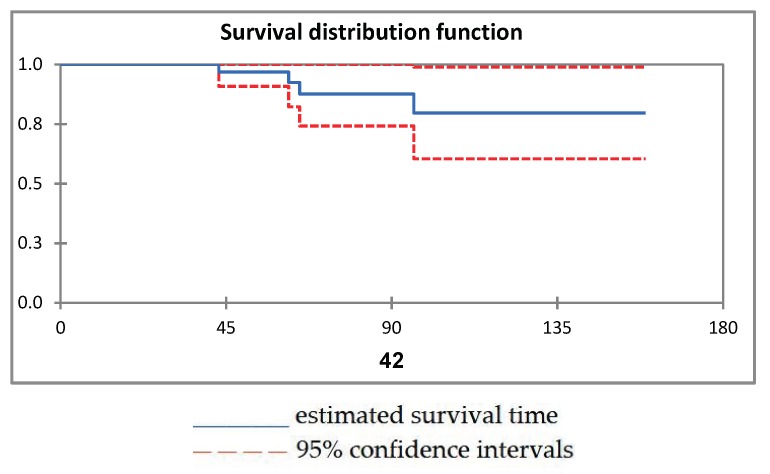
Kaplan Meier survival analysis.

Kaplan Meier survival analysis showed a survival of 90% at 96 months of follow up (95% CI, 60–90) (Figure 2). 

## 4. Discussion

Previous studies suggest a high incidence of complications following the revision of hemiarthroplasty to a THR, particularly dislocation and early loosening [3,11]. In our case series, the patients improved significantly following the conversion. The current trend is that careful selection of patients ameliorates the outcome of arthroplasty (hemiarthroplasty or THR) for patients with femoral neck fractures [9,11]. 

Furthermore, hemiarthroplasty of the hip is still a very common orthopaedic operation especially in elderly people for sub-capital neck of femur fractures [2,3]. Early conversion to THR in patients with painful hemiarthroplasty is also proposed [15].

There are limited reports about the ratio of conversion of hemiarthroplasty to THR. With regards to the conversion surgery, one of the common reasons is groin pain, although it does not help for relieving the pain fully in every patient [3,16]. Nevertheless, conversion of a painful hemiarthroplasty to THR has a good result for both pain relief and function [3,17]. In our series, the pain level improved by a mean of 2.37 and functionally, the mean improvement of the walking score was 1.95.

However, several studies showed high incidence of intra- and post-operative complications with conversion of a hemiarthroplasty to THR [3,11,17,18]. Those most commonly include proximal femoral fractures, perforations of the medial cortex with stem protrusion, instability, infection, deep venous thrombosis, and progressive loosening. Sierra and Cabanela reported the results of a large case series with 132 hemiarthroplasties and the ratio of major complications was 45% [11]. Kofoed et al. reported a 37% incidence of conversion in 106 patients with femoral neck fractures within the first 2 years after the primary operation using the Austin-Moore prosthesis [19] and another study by Alazzawi et al. reported a revision rate of 1.2–4% at 5 years after hemiarthroplasty [20].

The literature draws a particular attention to increased dislocation rates [3,17,18]. Sah and Estok argued that in revision hip arthroplasty, dislocation risk is three to five times higher than primary hip arthroplasty [17,21]. In fact, conversion of hip hemiarthroplasty to a THR is a revision arthroplasty, but it differs in that a native acetabulum is replaced and the subsequent prosthetic femoral head is smaller [17].

The general perspective is that smaller heads are more prone to dislocation, however, a larger head does not guarantee stability [17,22,23] (Table 7). Amstutz et al. reported that 11% of the patients with THR who had large-diameter (size ≥ 36 mm) heads for recurrent dislocation and revision unrelated to dislocation, remained unstable after revision at a mean follow-up of 5.5 years and subsequently underwent re-revision [22,23]. Furthermore, in their study, Sah et al. described dislocations even with 40-mm head size in revision surgery. Blumenfeld and Bargar hypothesized that increasing the femoral head diameter and revising the liner of a recurrently dislocating THR will not always be successful [24]. Therefore, a larger femoral head does not ensure stability and increasing the size of the femoral head is not the only way to reduce the rate of dislocation; optimal alignment of the components and repair of the posterior capsule could achieve a similar effect [25].

CHR in our orthopaedic hospital is the start point of modern total hip replacement with a success story and the gold standard using a 22.225-mm head diameter. Our database showed less than 1% dislocation for primary CHR and 8–12% dislocation rates for revision surgery, which is similar to the international literatures estimating to occur in 1% to 3% for primary (THR) and in 7% to 10% of revision surgeries, or even up to 28% [17,21,24]. Table 7 shows the literature with similar subjects; conversion of hemiarthroplasty to total hip replacement with up to 45% having complications and 14.3% revision rate. Our study with a head size of 22 shows similar results in terms of complications and particularly dislocation rates to the other studies. 

Certain centers are still using a 22-mm head with a high success and low dislocation rate in primary hip replacement [25,29] and for example, the Norwegian Arthroplasty Register showed a higher number of hip replacements with a 22-mm head than any other head size [30]. Also, Dutch Arthroplasty Register showed that femoral head sizes of 22 to 28 mm and 32 mm had a comparable risk of revision for any reason except dislocation, while 36-mm femoral head THAs had a 16% increased risk [31]. Byström stated that the Charnley technique may have become so well established among some surgeons over a long period that the rate of intraoperative technical errors is lower than with other types of prostheses [27]. That is why we believe that this study will be of interest to orthopedic surgeons.

Limitation: The number of patients included was limited, however, the articles in the literature regarding conversion of hemiarthroplasty to total hip and their case numbers were also limited

## 5. Conclusions

There was a tendency of the acetabulum to erode medially in the presence of infection. There was a significant improvement of the pain level and walking ability following conversion of hemi-arthroplasty to CHR. 

One of the main findings of the current study is that the dislocation rate with this technique was comparable to revision THR even though 22.225-mm heads were primarily used. A larger head might not be the answer to decrease the dislocation rate. Complication rates during the revision of hip hemiarthroplasty to modern CHR with 22.225-mm head diameter were comparable to first-time THR revision despite a smaller head. A longer follow up of the patients in this study may give us more valuable information about the outcome and survivorship of this particular implant.

## Figures and Tables

**Table 1:** Charnley’s modified pain and mobility scoring system.

Charnley Scoring System—Pain
6—No Pain
5—Slight or intermittent. Pain on starting to walk but getting less with normal activity
4—Pain only after some activity; disappears quickly with rest
3—Pain tolerable permitting limited activity
2—Severe pain on attempting to walk. Prevents all activity
1—Severe spontaneous
Charnley Scoring System—Walk
6—No walking aid
5—No stick but a limp
4—Long distance with one stick; limited without a stick
3—Limited with one stick. Difficult without stick. Able to stand long period
2—Time and distance very limited with or without sticks
1—Bedridden or few yards; two sticks or crutches

**Table 2:** Hodgkinson’s classification of Roentgenographic Demarcation of the socket.

Type 0	No demarcation at the cement bone interface
Type 1	Demarcation (1 mm) of outer one-third only
Type 2	Demarcation (1 mm) of outer one-third and middle one-third
Type 3	Complete demarcation (1 mm)
Type 4	Socket migration (change of socket position as judged on serial roentgenograms)

**Table 3:** Type of hemaiarthroplasties in primary operations.

Implant Type	Cemented Thompson	Austin-Moore	Bi-polar	Charnley Hasting	Unknown	Uncemented Thompson
Number of patients	17	15	5	2	2	1

**Table 4:** Hemiarthroplasty complications.

Hemiarthroplasty Complications	Number of Patients in Each Group and (%) in Bracket
Acetabular erosion	26 (62)
Stem loosening	10 (24)
Periprosthetic fracture	1 (2.4)
Deep infection	10 (24)
Wound infection	2 (4.8)
DVT	4 (9.6)

DVT: deep vein thrombosis.

**Table 5:** Direction of erosion and infections.

Unclear Direction		Medial Wall Erosion		Superior Wall Erosion		Both Medial and Superior Wall Erosion	
Infection	No Infection	Infection	No Infection	Infection	No Infection	Infection	No Infection
1	6	4	3	1	6	4	1

**Table 6:** Complications following conversion surgeries.

Post-Revision Complications	Dislocation	Infection	Wound Dehiscence	Trochanteric Non-Union	Trochanteric Bursitis	Total
Percent (number of patients)	9.5(4)	7.1(3)	4.8(2)	4.8(2)	2.4(1)	28.6(12)

**Table 7:** Publications in the literature on revision of hemi-arthroplasty to total hip replacement.

Publication	Case Number	Mean Follow-Up in Years	Survivorship	Complication Rate: *n* (%)	Dislocation Rate: *n* (%)	Revision (Due to Instability): *n* (%)	Head Size
**A-** **Figved (national registry) [26]**	473	5.8	93% at 5 years	48 (10%)		39 (8.25%)	22(219), 28 (138), 30(16), 32(44)
**B-Pankaj [3]**	44	6.4	97.4% at 72 months	10 (22.7%)	1 (2.27%)		
**C-Sierra [9]**	132	7.1	96.5% at 5 years	59 (45%)	13 (9.8%)	9 (6.8%)	
**D-Mounsey [27]**	28	5.8		10 (35.7%)	1 (3.6%)	4 (14.3%)	
**E-Diwanji [28]**	25	7.2		6 (24%)	4 (16%)	1 (4%)	
**F-Our study**	42	6.3	90% at 96 months	8(19%)	4 (9.5%)	2 (4.8%)	22
